# Phase 1 dose escalation study of FGFR4 inhibitor in combination with pembrolizumab in advanced solid tumors patients

**DOI:** 10.1002/cam4.5532

**Published:** 2023-01-09

**Authors:** Jianming Xu, Jiuwei Cui, Haiping Jiang, Yan Zeng, Xiuyu Cong

**Affiliations:** ^1^ Oncology Department, Chinese PLA General Hospital Beijing China; ^2^ Oncology Department The First Hospital of Jilin University Changchun China; ^3^ Oncology Department The First Affiliated Hospital of Zhejiang University Hangzhou China; ^4^ EverNov Medicines (Zhuhai Hengqin) Co., Ltd Zhuhai China

**Keywords:** FGF19, FGFR4, pembrolizumab, Phase 1, solid tumor

## Abstract

**Objective:**

Inhibition of fibroblast growth factor (FGF) 19‐FGF Receptor 4 (FGFR4) signaling demonstrates potent anticancer activity. EVER4010001 is a highly selective FGFR4 inhibitor and pembrolizumab is approved for the treatment of several solid tumors. This study determined the maximum tolerated dose (MTD), recommended Phase 2 dose (RP2D), pharmacokinetics, safety, and preliminary efficacy of EVER4010001 plus pembrolizumab in patients with advanced solid tumors.

**Methods:**

This Phase 1, multicenter, open‐label study enrolled 19 Asian–Chinese patients (57.9% male: median age 58 years) with advanced solid tumors. For “3+3” dose escalation, 3–6 patients received treatment at each dose level (EVER4010001 40, 60, 80, or 100 mg twice daily [BID] plus pembrolizumab 200 mg every 3 weeks).

**Results:**

At the data cutoff (August 12, 2021), no dose‐limiting toxicities (DLTs) were reported at 40 mg–80 mg. At 100 mg, 2 (40.0%) patients had 3 DLTs within the 28‐day DLT observation period after first administration. Median time to peak EVER4010001 concentration (*T*_max_) was 0.55–1.03 hours. Mean terminal EVER4010001 half‐life (T_1/2_) was 4.00–4.92 hours. The area under the concentration‐time curve (AUC_0–t_) and maximum observed concentration (*C*_max_) ranged from 2370.87–5475.77 hour*ng/ml and 606.07–1348.86 ng/ml, respectively. The most common EVER4010001‐related treatment‐emergent adverse events were diarrhea (94.7%), increased aspartate aminotransferase (57.9%), and increased alanine aminotransferase (47.4%).

**Conclusion:**

Eighty milligrams BID was the MTD and RP2D for EVER4010001 plus pembrolizumab. Efficacy results were promising, and no new safety risks were reported, justifying the Phase 2 portion of this study.

## Graphical abstract

Inhibition of fibroblast growth factor 19‐FGF Receptor 4 signaling demonstrates potent anticancer activity. EVER4010001 is a highly selective FGFR4 inhibitor and pembrolizumab is approved for the treatment of several solid tumors. This study determined the maximum tolerated dose (MTD), recommended Phase 2 dose (RP2D), pharmacokinetics, safety, and preliminary efficacy of EVER4010001 plus pembrolizumab in patients with advanced solid tumors.

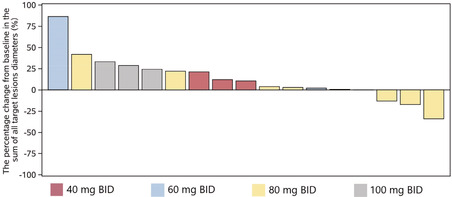

## 1 INTRODUCTION

Despite numerous prevention and control efforts, cancer is still a major health problem and one of the leading causes of death worldwide.^1^, ^2^ The global cancer burden is further expected to rise by 47% in the next 20 years, reaching 28.4 million cases in 2040.^2^ Globally, the leading causes of cancer‐related death are lung, colorectal, liver, stomach, and breast cancers.^1^, ^3^

Fibroblast growth factors (FGFs) are structurally related polypeptides with diverse biological activities.^4^ Most FGFs bind and activate an FGF receptor (FGFR), initiating multiple signaling cascades.^5^ FGF‐FGFR signaling plays an important role in development and tissue repair by regulating cellular processes such as growth, differentiation, migration, morphogenesis, and angiogenesis.^6^, ^7^ Dysregulation of this signaling network is important for tumor development, and aberrant FGFR signaling (i.e., amplification, overexpression, mutation, and translocation) has been characterized in almost all cancer types.^6^, ^8^ Of the four FGFRs (FGFR1–4), the function of the FGFR4 signaling pathway in cancer is the least characterized, although it is known to play an important role in hepatobiliary physiology and tumor progression.^6^, ^7^, ^9^, ^10^, ^11^, ^12^ FGFR4 overexpression has been reported in several solid tumors, including hepatocellular carcinoma (HCC), oropharyngeal squamous cell carcinoma, breast cancer, and pancreatic cancer.^13^

FGF19 is an important regulator of metabolism under normal physiological conditions.^6^ It signals through FGFR4 and its co‐receptor β‐klotho (KLB) to play a key role in the regulation of bile acid homeostasis, and has been associated with liver tumorigenesis.^6^, ^13^ The activation of FGF19‐FGFR4 signaling has recently been closely associated with cancer development and progression, suggesting this may be an attractive target for effective anticancer therapeutics.^9^, ^14^, ^15^ Therefore, there is increasing interest in therapeutically inhibiting the FGF19‐FGFR4 signaling axis in FGFR4/KLB‐positive solid tumors, especially in the HCC setting.^16^, ^17^

In preclinical studies, inhibition of the FGF19‐FGFR4 signaling pathway has demonstrated antitumor activity and prolonged overall survival (OS) in mice with high‐expressing FGF19/FGFR4/KLB tumors at well tolerated doses.^18^, ^19^, ^20^, ^21^, ^22^

EVER4010001 (FGF401, roblitinib) is a highly selective and potent FGFR4 inhibitor, which binds the kinase domain of the adenosine triphosphate (ATP) binding‐site of FGFR4.^23^ In kinase biochemical assays, EVER4010001 inhibited FGFR4 with a half‐maximal inhibitory concentration (IC_50_) of 2.4 Nm, with >1000‐fold selectivity against all other kinases (including FGFR1, FGFR2, and FGFR3).

Other FGFR4 inhibitors such as BLU‐554 and H3B‐6527 have been investigated in Phase 1 and Phase 1/2 clinical trials for HCC and displayed potent anticancer abilities.^13^, ^24^, ^25^, ^26^, ^27^

A Phase 1/2 open‐label study (CFGF401X2101 trial; NCT02325739) evaluating the safety and efficacy of FGF401 in patients with HCC or solid tumors with positive FGFR4 and KLB expression was the precursor to this study.^28^ Daily (QD) doses between 50 and 150 mg were administered to fasted or unfasted patients, with 120 mg QD determined as the recommended Phase 2 dose (RP2D). FGF401 alone or combined with the anti‐programmed cell death‐1 (PD‐1) antibody spartalizumab was safe in patients with FGFR4/KLB‐positive tumors, with preliminary clinical efficacy observed.

Pembrolizumab is a humanized monoclonal immunoglobulin G4 kappa anti−PD‐1 antibody which has been tested clinically in a series of KEYNOTE studies, with promising effects.^29^, ^30^, ^31^, ^32^, ^33^ Consequently, the Food and Drug Administration has approved pembrolizumab for the treatment of adult and pediatric patients with unresectable or metastatic tumor mutational burden‐high solid tumors.^34^ Pembrolizumab is still being investigated in several categories of malignancies through a series of KEYNOTE trials (bladder, breast, colorectal, esophagus, gastric, head/neck, lung, melanoma, ovarian, and other solid tumors) to further determine its clinical efficacy.^29^, ^35^

The combination of EVER4010001 and pembrolizumab in the clinical setting has not previously been investigated. However, the combined use of pembrolizumab with another FGF inhibitor, lenvatinib, has previously been studied. By blocking FGFR4, lenvatinib has reduced tumor programmed death ligand 1 (PD‐L1) levels to improve anti‐PD‐1 efficacy.^36^ The gene *FGFR4* is involved in a distinct 8‐gene mammalian target of rapamycin (mTOR) signature which is associated with increased PD‐1/PD‐L1 expression, and consequently, was predictive of better survival in patients upon immune checkpoint inhibitor (ICI) treatment in multiple cancers.^37^ This is further supported by a preclinical trial that found lenvatinib plus an anti‐PD‐1 ICI activated immune pathways and could benefit ~20% of patients with HCC.^38^ Results from the Phase 1b KEYNOTE‐524 trial showed lenvatinib plus pembrolizumab had promising antitumor activity and manageable toxicities in patients with HCC.^39^ Lenvatinib plus pembrolizumab also led to significantly longer progression‐free survival and OS than standard care in Phase 3 trials in patients with advanced endometrial cancer and advanced renal cell carcinoma.^40^, ^41^

Only the results from the Phase 1 dose escalation portion of this Phase 1/2 study are described here, where the safety of EVER4010001 twice daily (BID) treatment combined with pembrolizumab was evaluated in patients with advanced solid tumors to identify the maximum tolerated dose (MTD) and RP2D. We further evaluated the pharmacokinetic (PK) profile, safety, and efficacy of the treatment regimen.

## 2 MATERIALS AND METHODS

### 2.1 Study participants

Eligible patients were males or females ≥18 years of age with a histologically or cytologically confirmed metastatic or locally advanced solid tumor, for which no effective standard therapy exists, or had previously failed treatment. Eligible patients had at least one measurable lesion according to Response Evaluation Criteria in Solid Tumors (RECIST) v1.1 and an Eastern Cooperative Oncology Group (ECOG) performance status ≤1. Patients were not previously treated with FGF19‐FGFR4 therapy/pan‐FGFR inhibitor. Eligible patients had no impairment of gastrointestinal function/ongoing active diarrhea/irritable bowel syndrome that could alter EVER4010001 absorption. All patients had provided written informed consent.

### 2.2 Study design

This was a Phase 1/2, multicenter, open‐label study conducted at over 33 study sites (3 sites for Phase 1) across China, in patients with advanced solid tumors for the Phase 1 dose escalation component (the focus of this manuscript), followed by a Phase 2 indication expansion part (ongoing). The primary study objective for the Phase 1 part was to determine the MTD and RP2D of EVER4010001 in combination with pembrolizumab. The secondary objectives were to characterize the PK and individual drug exposure, assess anti‐tumor efficacy, safety, and tolerability of EVER4010001 with pembrolizumab, and explore predictive biomarkers.

In the Phase 1 part, EVER4010001 was administered orally at a starting dose of 40 mg BID, with pembrolizumab 200 mg administered via intravenous infusion on Day 1 of each 21‐Day treatment cycle (Q3W). After a screening period of up to 28 days, eligible patients received treatment according to a standard “3+3” dose escalation design. Depending on the occurrence of dose‐limiting toxicities (DLTs), 3–6 patients were enrolled at each dose level (EVER4010001 40, 60, 80, or 100 mg BID plus pembrolizumab 200 mg Q3W). Treatment continued until unacceptable toxicity, progressive disease, and/or at the discretion of the Investigator or patient's withdrawal of consent.

### 2.3 Study assessments and endpoints

The primary endpoint was DLT observed within 28 days of the first dose of EVER4010001 in combination with pembrolizumab. Secondary endpoints included PK parameters (e.g., maximum observed concentration [*C*_max_] and area under the concentration‐time curve [AUC]). Secondary endpoints included objective response rate (ORR), disease control rate (DCR), and duration of response (DOR) as assessed by investigator per RECIST v1.1. Tumor responses were assessed according to RECIST v1.1; computerized tomography scanning or magnetic resonance imaging was performed every 6 weeks in the first 48 weeks, and every 12 weeks thereafter. Secondary endpoints also included safety and tolerability (adverse events [AEs] as defined by the National Cancer Institute‐Common Terminology Criteria for Adverse Events CTCAE v5.0) and exploratory biomarkers (total bile acids, 7‐α‐hydroxy‐4‐cholesten‐3‐one [C4] and circulating FGF19). Safety follow‐up visit was performed within 30 days (±5 days) and 90 days (±7 days) after the last dose of the study treatment.

### 2.4 Statistical analyses

All patients who received at least one dose of the study treatment in the Phase 1 part of the study were included in the safety and efficacy analyses. Dose escalation and MTD assessment were primarily based on DLTs which occurred within the 28‐day observation period. Descriptive statistics were used for demographics, safety, and efficacy data. The number of patients who experienced DLTs (within and outside of the 28‐day DLT assessment period) was tabulated for each dose level. The sample size of patients required for dose escalation were determined based on the ‘3+3’ design and DLTs observed as the trial progressed.

## 3 RESULTS

### 3.1 Study participants

A total of 19 patients were treated with EVER4010001 BID (3, 3, 6, and 7 patients dosed with 40, 60, 80, and 100 mg, respectively), of which 2 patients in the 100 mg EVER4010001 BID group were replaced and not included in determination of MTD. Most patients were male (57.9%), and all patients were of Asian‐Chinese origin and Han ethnicity (Table 1). The median age was 58 years (range 28–75 years). The ECOG performance status was assessed as 0 for 7 (36.8%) patients, with the remainder assessed as 1. Patients presented with nine different cancer types, with the most common being lung (7 [36.8%]), pancreatic (3 [15.8%]), and rectal cancers (3 [15.8%]).

**TABLE 1:** Demographics and baseline disease characteristics

Characteristics and statistics	Patients (*N* = 19)
Median age, years (range)	58.0 (28.0–75.0)
Gender, *n* (%)	
Female	8 (42.1)
Male	11 (57.9)
Race, *n* (%)	
Asian‐Chinese	19 (100.0)
Other	0
Baseline ECOG performance status, *n* (%)	
0	7 (36.8)
1	12 (63.2)
Cancer type, n (%)	
Biliary tract	1 (5.3)
Breast	1 (5.3)
Cholecyst	1 (5.3)
Duodenal	1 (5.3)
Lung	7 (36.8)
Pancreas	3 (15.8)
Rectum	3 (15.8)
Stomach	1 (5.3)
Uterus	1 (5.3)

Abbreviation: ECOG, Eastern Cooperative Oncology Group.

At the time of data cut‐off (DCO) on August 17, 2021, 3 (15.8%) patients were still ongoing with study medication, 16 (84.2%) patients had discontinued. A total of 12 (63.2%) patients discontinued due to disease progression, 3 (15.8%) due to AEs, and 1 (5.3%) due to physician's decision (Table S1). The mean total treatment duration of EVER4010001 was 93.4 (±109.89) days (Table S2). The mean total actual dose of EVER4010001 was close to the total intended dose (actual 13246.3 mg vs. intended 13385.3 mg), indicating that the frequency of dose interruptions and reductions had a limited impact on the exposure of EVER4010001. The mean relative dose intensity of EVER4010001 was 97.18% (±4.780%). Only 1 (5.3%) patient, in the 100 mg BID group, had a dose reduction twice because of increased alanine aminotransferase (ALT) and aspartate aminotransferase (AST).

### 3.2 DLT

No DLTs were reported for EVER4010001 40–80 mg BID groups (Table S3). In the 100 mg BID group (*N* = 5), two (40.0%) patients had three DLT events within 28 days of first administration (two cases of increased AST and one case of increased ALT), of which one patient had an event of increased AST beyond 28 days. Based on the obtained safety and PK data, the Safety Monitoring Committee determined that 80 mg BID was the MTD and RP2D for EVER4010001 when used in combination with pembrolizumab (200 mg, Q3W).

### 3.3 PK parameters and exploratory biomarkers

**FIGURE 1:**
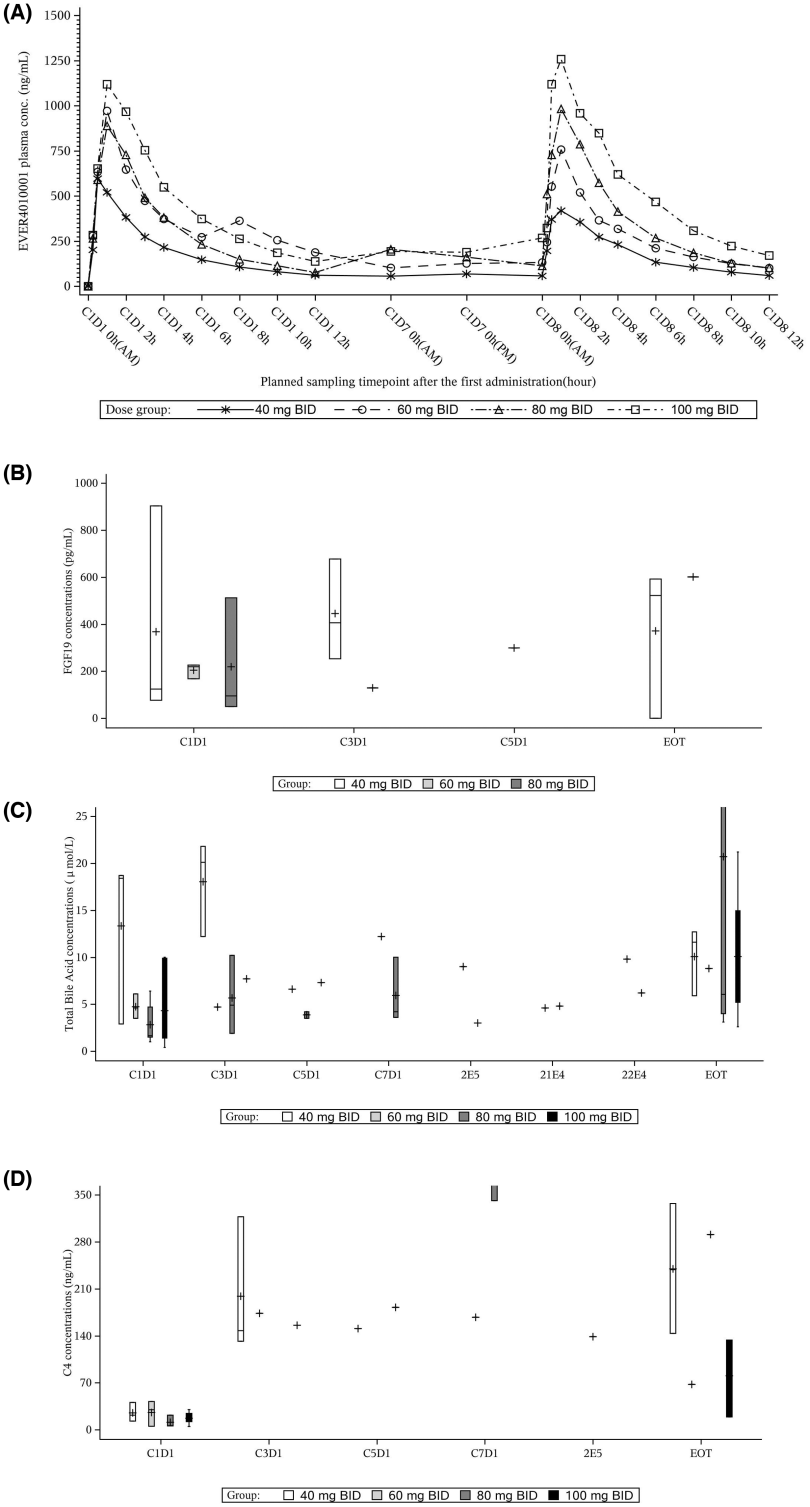
The horizontal line represents the median, the box represents quartile 1 through quartile 3, and the +symbol represents the mean. Whiskers extend to the most extreme observation within 1.5 times the interquartile range from the nearest quartile, so that all outliers >1.5 times the interquartile range are individually displayed. (A) EVER4010001 plasma concentration‐time plot (linear scale). (B) Boxplots of plasma FGF19 concentrations by visit. (C) Boxplots of plasma total bile acid concentrations by visit. (D). Boxplots of plasma C4 concentrations by visit. BID, twice daily; C, cycle; C4, 7‐α‐hydroxy‐4‐cholesten‐3‐one; D, day; FGF19, fibroblast growth factor 19.

The median time to peak EVER4010001 concentration (*T*_max_) was 0.55 to 1.03 h. (Table 2). The mean plasma terminal half‐life (*T*_1/2_) of EVER4010001 ranged from 4.00 to 4.92 h. Both *T*_max_ and *T*_1/2_ generally appeared independent of dose levels. The geometric means of AUC_0–*t*_ and *C*_max_ were in the range of 2363.4 to 5459.69 h*ng/ml and 559.90 to 1337.29 ng/ml, respectively, at Cycle 1 Day 1, with low to moderate variation between patients. Based on *T*_1/2_, the steady state was reached before Cycle 1 Day 8 after repeated dosing. The observed AUC_0–*t*_ and *C*_max_ values on Cycle 1 Day 8 were comparable to those on Cycle 1 Day 1, with the accumulation ratio (*R*_ac_) around 1. Treatment elevated C4, total bile acid concentration, and circulating FGF19 in most patients treated with 40, 60, 80, and 100 mg EVER4010001 (Figure 1).

**TABLE 2:** Summary of PK parameters for EVER4010001

PK parameters	40 mg BID (*N* = 3)	60 mg BID (*N* = 3)	80 mg BID (*N* = 6)	100 mg BID (*N* = 7)
Geometric mean AUC_0–*t*_, Hour·ng/ml (geometric CV%)				
Cycle 1, Day 1	2363.64 (9.59)	4464.03 (37.96)	3774.38 (14.53)	5459.69 (8.34)
Cycle 1, Day 8	2084.60 (35.99)	3212.66 (27.61)	4284.35 (25.05)	6214.09 (24.13)
Geometric mean *C*_max_, ng/ml (geometric CV%)				
Cycle 1, Day 1	559.90 (17.80)	958.22 (19.91)	897.03 (23.16)	1337.29 (14.59)
Cycle 1, Day 8	426.59 (65.76)	748.76 (18.25)	991.13 (30.14)	1329.72 (22.28)
Mean *t*_1/2_, Hour (SD)				
Cycle 1, Day 1	4.92 (0.16)	4.00 (0.36)	4.64 (1.44)	4.22 (0.69)
Cycle 1, Day 8	4.90 (2.59)	5.78 (1.12)	4.12 (0.90)	4.97 (0.55)
Median *t*_max_, Hour (Min, Max)				
Cycle 1, Day 1	0.550 (0.52, 1.05)	0.970 (0.93, 1.05)	1.030 (0.23, 2.00)	1.000 (0.52, 2.05)
Cycle 1, Day 8	1.030 (0.57, 2.00)	0.970 (0.93, 1.07)	0.990 (0.25, 1.05)	0.970 (0.50, 2.97)
Mean *R*_ac_ (SD)				
Cycle 1, Day 8	1.24 (0.25)	1.31 (0.11)	1.16 (0.08)	1.23 (0.05)

Abbreviations: AUC, area under curve; BID, twice daily; *C*_max_, maximum concentration; CV, coefficient of variation; PK, pharmacokinetic; *R*_ac_, drug accumulation ratio; SD, standard deviation; *T*_1/2_, half‐life; *T*_max_, time to reach maximum plasma concentrations.

### 3.4 Safety

All 19 patients experienced treatment‐emergent AEs (TEAEs), of which most were of CTCAE Grade 1 or 2. A total of 19 (100%) and 18 (94.7%) patients reported TEAEs related to EVER4010001 or pembrolizumab, respectively. All 19 patients experienced TEAEs of gastrointestinal disorders; the most common was diarrhea (18/19 patients, 94.7%) (Table S4). The most common TEAEs related to EVER4010001 by preferred term (PT) were diarrhea (18 [94.7%]), increased AST (11 [57.9%]), and increased ALT (9 [47.4%]). The most common TEAEs related to pembrolizumab by PT were diarrhea (11 [57.9%]), increased AST (10 [52.6%]), and increased ALT (8 [42.1%]). The most common TEAEs related to EVER4010001 and pembrolizumab by (PT) were diarrhea (11 [57.9%]), increased AST (10 [52.6%]), and increased ALT (8 [42.1%]; Table 3). No Grade 4 or 5 TEAEs related to EVER4010001 and/or pembrolizumab were reported. More EVER4010001‐related TEAEs were observed in the 100 mg BID group versus the other three dosing groups. For example, proteinuria (42.9%), anemia (42.9%), hyperphosphatemia (57.1%), and increased blood alkaline phosphatase (42.9%) (Table S5).

**TABLE 3:** Most common EVER4010001 and pembrolizumab‐related TEAEs of any grade (observed in ≥10% of patients) or grade ≥3 (observed in ≥5% of patients)

SOC	Patients *n* (%) (*N* = 19)	
PT	Any grade	Grade ≥3
Any EVER4010001 and pembrolizumab‐related TEAEs^a^		
Blood and lymphatic system disorders	4 (21.1)	0
Anemia	4 (21.1)	0
Gastrointestinal disorders	11 (57.9)	1 (5.3)
Abdominal pain	2 (10.5)	0
Diarrhea	11 (57.9)	1 (5.3)
Investigations	14 (73.7)	2 (10.5)
Alanine aminotransferase increased	8 (42.1)	1 (5.3)
Aspartate aminotransferase increased	10 (52.6)	2 (10.5)
Blood alkaline phosphatase increased	4 (21.1)	0
Blood bilirubin increased	3 (15.8)	0
Gamma‐glutamyl transferase increased	2 (10.5)	0
Metabolism and nutrition disorders	6 (31.6)	0
Hyperphosphatemia	3 (15.8)	0
Hypoalbuminemia	3 (15.8)	0
Renal and urinary disorders	4 (21.1)	0
Proteinuria	4 (21.1)	0
Skin and subcutaneous tissue disorders	4 (21.1)	0

Abbreviations: MedDRA, Medical Dictionary for Regulatory Activities; PT, preferred term; SOC, system organ class; TEAE, treatment‐emergent adverse event.

^a^ Data were coded using MedDRA V24.0.

A total of 9 (47.4%) patients reported Grade ≥3 TEAEs, of which 3 (15.8%) were related to both EVER4010001 and pembrolizumab. The remaining six patients reported Grade ≥3 TEAEs unrelated to EVER4010001 or pembrolizumab which were not confined to a small group of PTs. The most common Grade ≥3 TEAEs by PT were increased ALT (2 [10.5%]), increased AST (2 [10.5%]), and increased blood bilirubin (2 [10.5%]) (Table S4).

A total of 4 (21.1%) patients experienced TEAEs which led to discontinuation of EVER4010001 and pembrolizumab due to increased AST (2 [10.5%]), increased blood bilirubin (1 [5.3%]), and superior vena cava syndrome (19 [5.3%]) (Table S5). Patients experienced TEAEs leading to temporary interruption of pembrolizumab (3 [15.8%]), including 1 (5.3%) patient each with thyroiditis, increased ALT, and increased blood bilirubin; EVER4010001 (4 [21.0%]), where reasons for dose interruption were not confined to a single/small group of PTs; and EVER4010001 and pembrolizumab (2 [10.5%]).

Only 2 (10.5%) patients (40 and 60 mg) experienced serious AEs (SAEs); none were related to EVER4010001 or pembrolizumab. SAEs by PT included pancreatitis, device dislocation, and a death of unknown cause (Table S5). A total of 7 (36.8%) patients experienced immune‐related AEs during the study.

### 3.5 Efficacy

**FIGURE 2:**
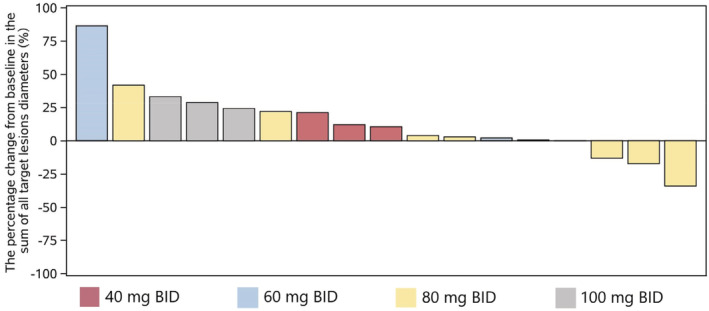
Waterfall plot showing the percentage of change in sum of target lesions from baseline by patients and treatment dose. BID, twice daily.

The study examined the efficacy of EVER4010001 in combination with pembrolizumab across four dosing levels (Figure 2). ORR was 16.7% in the 80 mg BID group, due to one patient achieving a partial response (Table 4). In the 40, 60, 80, and 100 mg BID groups, DCR was 66.7% (2/3), 33.3% (1/3), 50% (3/6), and 0% (0/7), respectively. No mature DOR data was available before the cut‐off date because the patient with PR had not yet experienced PD.

**TABLE 4:** Summary of treatment efficacy

Efficacy	40 mg BID (*N* = 3)	60 mg BID (*N* = 3)	80 mg BID (*N* = 6)	100 mg BID (*N* = 7)
Objective response (CR+PR), n (%)	0	0	1 (16.7)	0
95% CI	0.2924, 1.0000	0.2924, 1.0000	0.0042, 0.6412	0.5407, 1.0000
Best overall response, n (%)				
Complete response	0	0	0	0
Partial response	0	0	1 (16.7)	0
Stable disease	2 (66.7)	1 (33.3)	2 (33.3)	0
Progressive disease	1 (33.3)	2 (66.7)	2 (33.3)	5 (71.4)
Not evaluable	0	0	1 (16.7)	1 (14.3)
Disease control rate (CR+PR+SD), n (%)	2 (66.7)	1 (33.3)	3 (50.0)	0
95% CI	0.0943, 0.9916	0.0084, 0.9057	0.1181, 0.8819	0.5047, 1.0000

Abbreviations: BID, twice daily; CI, confidence interval; CR, complete response; PR, partial response; SD, stable disease.

## 4 DISCUSSION

FGF‐FGFR signaling dysregulation is reported in a wide range of cancers, specifically the FGF19‐FGFR4 pathway plays a pivotal role in cancer initiation and progression.^7^, ^9^, ^42^, ^43^ Preclinical data has shown promising results for the use of EVER401001, where it induced regression/stasis in triple positive HCC tumors, supporting its use in clinical trials.^16^, ^17^, ^18^, ^29^

The MTD and RP2D was declared at 80 mg BID for EVER4010001 when used in combination with pembrolizumab (200 mg, Q3W) and will be evaluated further in the indication expansion (Phase 2) part of this study. In the CFGF401X2101 trial, RP2D was established as 120 mg QD for FGF401 in combination with spartalizumab (300 mg, Q3W).^44^ PK/PD studies have shown that the pharmacodynamics and efficacy of EVER4010001 are related to the steady‐state trough plasma concentration, and that plasma exposure increases with doses. Considering the half‐life of EVER4010001, drug exposure is more stable with a BID dosing regimen compared to QD, and the steady state trough concentration can be better maintained at a high level. Therefore, it was hypothesized that modifying the dosing schedule from QD to BID would stabilize the blood concentration of EVER4010001 (lower *C*_max_ and higher *C*_trough_ [lowest concentration of drug before the next dose]), improving tolerance and efficacy. At the RP2D of 80 mg BID in this study, the geometric mean *C*_max_ (geometric coefficient of variation %) was 991.13 ng/ml (30.14%) for EVER4010001 at Cycle 1 Day 8, compared to 1120 ng/ml (36.5%) at the RP2D (120 mg FGF401 QD) in the CFGF401X2101 trial.^44^ Drug exposures increased with dose from 40 to 100 mg and the inter‐patient variability of drug exposure was moderate. This is consistent with the CFGF401X2101 trial where FGF401 was well absorbed and drug exposure increased with dose proportionally with limited drug accumulation.^28^

Analysis of serum biomarkers (C4, total bile acid concentration, and circulating FGF19) were indicative of FGFR4 pathway suppression being achieved at all doses, therefore preliminary assessment indicated a favorable PK profile. The number of patients was also limited, therefore further research is needed to determine dose response and analyze the pharmacodynamics of EVER4010001; this will be evaluated in the Phase 2 portion of the study along with further PK investigations and biomarker analysis.

In the 80 mg group, 1/6 (16.7%) patients experienced a partial response, with ORR and DCR at 16.7% and 50.0%, respectively. This is similar to the CFGF401X2101 trial, where DCR was reported as 50.0% in each cohort receiving combined FGF401 and spartalizumab therapy.^44^ The ORR was 5.3% across all doses, which was similar to that reported in the CFGF401X2101 trial (8%).^28^, ^44^ The Phase 1 dose escalation portion of this study enrolled patients with non‐selective solid tumors to define the MTD and RP2D for EVER4010001, efficacy will further be assessed in the Phase 2 indication expansion portion of the study in target populations, e.g., patients with HCC.

Preliminary clinical data suggest EVER4010001 has a manageable safety profile that is consistent with FGF19 pathway inhibition and the safety profile reported in the CFGF401X2101 trial.^28^, ^44^ There were 4 and 3 DLT events during the study period and within 28 days after first administration, respectively. These DLT events were reported in two patients in the 100 mg BID group, with no DLTs reported for the 40–80 mg doses of EVER4010001.

Similarly, while the overall incidence of drug‐related TEAEs (irrespective of grade) was similar across the four dosing groups in this study, more EVER4010001‐related TEAEs were observed with the 100 mg BID group versus the other three dosing groups.

The most common TEAEs related to EVER4010001 (diarrhea, increased AST, and increased ALT) were the same as the CFGF401X2101 trial treating HCC and solid tumors with EVER4010001 monotherapy and EVER4010001 plus spartalizumab combination therapy.^28^, ^44^

Most AEs were Grade 1 or 2, with the most frequently observed AE, diarrhea, being an anticipated on‐target AE consistent with inhibition of the FGF19‐FGFR4 pathway and its role in bile synthesis.^22^, ^28^ The bile acid sequestrant cholestyramine was used in this study and the CFGF401X2101 trial to treat diarrhea and effectively, which further supports this hypothesis.^44^, ^45^ This is also consistent with the CFGF401X2101 trial, where 69% of patients experienced diarrhea suspected to be related to the study drug, compared to 57.9% in this study.^28^ Diarrhea is also commonly reported (~40% of cases) in lenvatinib treatment, the first‐line treatment for HCC, which inhibits FGFR1–4 amongst other receptors (e.g., vascular endothelial growth factor, platelet‐derived growth factor).^46^, ^47^, ^48^ Diarrhea has also previously been identified as a potential favorable prognostic factor for HCC disease progression during lenvatinib therapy, although this is not yet conclusive.^46^

In this study, increased AST was reported in 57.9% of patients as a TEAE related to EVER4010001, and in 10.5% of patients as a Grade ≥3 TEAE. This is similar to the CFGF401X2101 trial where increased AST was present in 47.5% of patients, and 18.8% of patients as a Grade ≥3 TEAE.^28^, ^44^ Here, increased ALT was reported in 47.5% of patients as a TEAE related to EVER4010001, and in 10.5% of patients as a Grade ≥3 TEAE. This is also similar to the CFGF401X2101 trial where increased ALT was reported in 43.8% of patients, and 15.0% of patients as a Grade ≥3 TEAE.^28^, ^44^ Transaminase elevations were likely on‐target effects of FGFR4 pathway inhibition.^44^, ^49^

The CFGF401X2101 trial also investigated FGF401 combined with the anti‐PD‐1 antibody, spartalizumab, where diarrhea, increased AST, and increased ALT were present in 58.3%, 50.0%, and 33.3% of the 12 patients, respectively.^44^ Despite a limited number of patients receiving combination therapy, the safety, tolerability, and efficacy was similar to FGF401 monotherapy in the CFGF401X2101 trial.

Overall, there were no significant changes in the knowledge of risks and benefits of EVER4010001 and pembrolizumab. Therefore, the overall risk‐benefit remains positive and justifies the continuation of the development program. EVER4010001 and the combination regimen will continue to be evaluated in the Phase 2 part of this study to further refine patient selection.

## AUTHOR CONTRIBUTIONS

**Jian‐Ming Xu:** Data curation (supporting); investigation (lead); methodology (lead); supervision (lead); writing – review and editing (lead). **Jiuwei Cui:** Data curation (supporting); investigation (lead); supervision (supporting); writing – review and editing (supporting). **Haiping Jiang:** Data curation (supporting); investigation (supporting); supervision (supporting); writing – review and editing (supporting). **Yan Zeng:** Investigation (supporting); methodology (lead); project administration (lead); supervision (lead); writing – original draft (lead). **Xiuyu Cong:** Data curation (lead); formal analysis (lead); investigation (supporting); methodology (lead); writing – review and editing (lead).

## FUNDING INFORMATION

Sponsored by EverNov Medicines (Zhuhai Hengqin) Co., Ltd.

## CONFLICT OF INTEREST

The authors have no conflict of interest. The study wasdesigned under the responsibility of EverNov Medicines. The study was funded by EverNov Medicines. EVER4010001 was provided by EverNov Medicines and pembrolizumab was provided by Merck Sharp & Dohme Corp. EverNov Medicines collected and analyzed the data and contributed to the interpretation of the study. All authors had full access to all of the data in the study and had final responsibility for the decision to submit for publication.

## ETHICS STATEMENT

*Approval of the research protocol by an Institutional Reviewer Board*: The study protocol and all amendments were reviewed by the Independent Ethics Committee or Institutional Review Board for each center. *Informed Consent*: All informed consent was obtained from the subjects and/or guardians. *Registry and the Registration No. of the study/trial*: NCT04699643. *Animal Studies*: N/A.

## Supporting information


Data S1

## Data Availability

The data that support the findings of this study are available from the corresponding author upon reasonable request.
