# Supplementary material for: Phase 1 dose escalation study of FGFR4 inhibitor in combination with pembrolizumab in advanced solid tumors patients
Source: Cancer Med. 2023 Jan 9;12(7):7762–71. doi: 10.1002/cam4.5532 (PMC10134273; doi:10.1002/cam4.5532)
Supplement: Supplementary file 1 — Data S1 [file CAM4-12-7762-s001.docx]

Phase 1 Dose Escalation Study of FGFR4 Inhibitor in Combination with Pembrolizumab in Advanced Solid Tumors Patients

**Authors’ names:**

Jianming Xu^1#^, Jiuwei Cui^2#^, Haiping Jiang^3^, Yan Zeng^4^, Xiuyu Cong^4^

^#^Contributed equally

# List of supporting information

Supplementary Table 1: Summary of Patient Disposition at DCO Across All Dose Levels

Supplementary Table 2: Summary of EVER4010001 Treatment Exposure Across All Dose Levels

Supplementary Table 3: Summary of DLTs Across All Dose Levels

Supplementary Table 4: Most Common EVER4010001-related TEAEs and Pembrolizumab‑related TEAEs of Any Grade (Observed in ≥10% of Patients) or Grade ≥3 (Observed in ≥5% of Patients)

Supplementary Table 5: Summary of Treatment Safety Across All Dose Levels

Supplementary Table 1: Summary of Patient Disposition at DCO Across all Dose Levels

| Safety | 40 mg BID (N=3) | 60 mg BID (N=3) | 80 mg BID (N=6) | 100 mg BID (N=7) | Total  (N=19) |
| --- | --- | --- | --- | --- | --- |
| **On-treatment, n (%)** | 0 | 1 (33.3) | 2 (33.3) | 0 | 3 (15.8) |
| **Discontinued EVER4010001 treatment, n (%)** | 3 (100.0) | 2 66.7%) | 4 (66.7) | 7 (100.0) | 16 (84.2) |
| Disease progression | 3 (100.0) | 2 (66.7) | 3 (50.0) | 4 (57.1) | 12 (63.2) |
| Adverse event | 0 | 0 | 1 (16.7) | 2 (28.6) | 3 (15.8) |
| Physician decision | 0 | 0 | 0 | 1 (14.3) | 1 (5.3) |
| **Dose reduction, n (%)** | 0 | 0 | 0 | 1 (14.3) | 1 (5.3) |

BID, twice daily; DCO, data cut-off

Supplementary Table 2: Summary of EVER4010001 Treatment Exposure Across All Dose Levels

| Exposure | 40 mg BID (N=3) | 60 mg BID (N=3) | 80 mg BID (N=6) | 100 mg BID (N=7) | Total  (N=19) |
| --- | --- | --- | --- | --- | --- |
| Mean total treatment duration, day (SD) | 91.3  (32.72) | 148.3  (200.85) | 134.2  (127.91) | 35.9  (41.88) | 93.4  (109.89) |
| Mean total actual dose, mg (SD) | 7293.3  (2596.10) | 17740.0  (24155.82) | 21306.7  (20449.64) | 6962.9  (8422.85) | 13246.3  (15864.31) |
| Mean total planned dose, mg (SD) | 7306.7  (2617.28) | 17800.0  (24102.48) | 21466.7  (20465.85) | 7171.4  (8375.70) | 13385.3  (15850.31) |
| Mean total relative dose intensity, % (SD) | 99.87  (0.23) | 98.17  (2.22) | 98.92  (1.08) | 94.13  (6.92) | 97.18  (4.78) |

BID, twice daily; SD, standard deviation

Supplementary Table 3: Summary of DLTs Across All Dose Levels

| DLT | 40 mg BID (N=3) | 60 mg BID (N=3) | 80 mg BID (N=6) | 100 mg BID (N=5) | Total  (N=17) |
| --- | --- | --- | --- | --- | --- |
| **Total DLTs within 28 days, n (%)** | 0 | 0 | 0 | 2 (40.0) | 2 (11.8) |
| Alanine aminotransferase increased | 0 | 0 | 0 | 1 (20.0) | 1 (5.9) |
| Aspartate aminotransferase increased | 0 | 0 | 0 | 2 (40.0) | 2 (11.8) |
| **Total DLTs during whole study, n (%)** | 0 | 0 | 0 | 2 (40.0) | 2 (11.8) |
| Alanine aminotransferase increased | 0 | 0 | 0 | 1 (20.0) | 1 (5.9) |
| Aspartate aminotransferase increased | 0 | 0 | 0 | 2 (40.0) | 2 (11.8) |

BID, twice daily; DLT, dose-limiting toxicity

Supplementary Table 4: Most Common EVER4010001-related TEAEs and Pembrolizumab‑related TEAEs of Any Grade (Observed in ≥10% of Patients) or Grade ≥3 (Observed in ≥5% of Patients)

| SOC | Patients n (%)  (N=19) | |
| --- | --- | --- |
| PT | Any Grade | Grade ≥3 |
| **Any TEAE^a^** | 19 (100) | 8 (42.1) |
| **Blood and lymphatic system disorders** | 9 (47.4) | 0 |
| Anemia | 9 (47.4) | 0 |
| **Gastrointestinal disorders** | 19 (100) | 3 (15.8) |
| Diarrhea | 18 (94.7) | 1 (5.3) |
| Investigations | 17 (89.5) | 4 (21.0) |
| Alanine aminotransferase increased | 9 (47.4) | 2 (10.5) |
| Aspartate aminotransferase increased | 11 (57.9) | 2 (10.5) |
| Blood bilirubin increased | 5 (26.3) | 2 (10.5) |
| **Metabolism and nutrition disorders** | 12 (63.2) | 1 (5.3) |
| Hypoalbuminemia | 11 (57.9) | 0 |
| **Any EVER4010001-related TEAEs** | 19 (100) | 3 (15.8) |
| **Blood and lymphatic system disorders** | 6 (31.6) | 0 |
| Anemia | 6 (31.6) | 0 |
| **Gastrointestinal disorders** | 18 (94.7) | 1 (5.3) |
| Abdominal pain | 2 (10.5) | 0 |
| Diarrhea | 18 (94.7) | 1 (5.3) |
| Nausea | 2 (10.5) | 0 |
| **Investigations** | 16 (84.2) | 2 (10.5) |
| Alanine aminotransferase increased | 9 (47.4) | 1 (5.3) |
| Aspartate aminotransferase increased | 11 (57.9) | 2 (10.5) |
| Blood alkaline phosphatase increased | 5 (26.3) | 0 |
| Blood bilirubin increased | 3 (15.8) | 0 |
| Gamma-glutamyl transferase increase | 3 (15.8) | 0 |
| Weight decreased | 2 (10.5) | 0 |
| **Metabolism and nutrition disorders** | 8 (42.1) | 0 |
| Hyperphosphatemia | 6 (31.6) | 0 |
| Hypoalbuminemia | 3 (15.8) | 0 |
| **Renal and urinary disorders** | 7 (36.8) | 0 |
| Proteinuria | 7 (36.8) | 0 |
| **Skin and subcutaneous tissue disorders** | 4 (21.1) | 0 |
| **Any pembrolizumab related TEAEs^a^** | 18 (94.7) |  |
| **Blood and lymphatic system disorders** | 4 (21.1) | 0 |
| Anemia | 4 (21.1) | 0 |
| **Endocrine disorders** | 3 (15.8) | 0 |
| Hypothyroidism | 3 (15.8) | 0 |
| Thyroiditis | 2 (10.5) | 0 |
| **Gastrointestinal disorders** | 11 (57.9) | 1 (5.3) |
| Abdominal pain | 2 (10.5) | 0 |
| Diarrhea | 11 (57.9) | 1 (5.3) |
| **Investigations** | 14 (73.7) | 2 (10.5) |
| Alanine aminotransferase increased | 8 (42.1) | 1 (5.3) |
| Aspartate aminotransferase increase | 10 (52.6) | 2 (10.5) |
| Blood alkaline phosphatase increased | 4 (21.1) | 0 |
| Blood bilirubin increased | 4 (21.1) | 0 |
| Gamma-glutamyl transferase increased | 2 (10.5) | 0 |
| **Metabolism and nutrition disorders** | 6 (31.6) | 0 |
| Hyperphosphatemia | 3 (15.8) | 0 |
| Hypoalbuminemia | 3 (15.8) | 0 |
| **Renal and urinary disorder** | 4 (21.1) | 0 |
| Proteinuria | 4 (21.1) | 0 |

MedDRA, Medical Dictionary for Regulatory Activities; PT, preferred term; SOC, system organ class; TEAE, treatment-emergent adverse event

^a^Data were coded using MedDRA V24.0

Supplementary Table 5: Summary of Treatment Safety Across All Dose Levels

| Safety | 40 mg BID (N=3) | 60 mg BID (N=3) | 80 mg BID (N=6) | 100 mg BID (N=7) | Total  (N=19) |
| --- | --- | --- | --- | --- | --- |
| **Immune-related events, n (%)** | 1 (33.3) | 1 (33.3) | 1 (16.7) | 4 (57.1) | 7 (36.8) |
| **EVER4010001-related TEAEs, n (%)** | 3 (100) | 3 (100) | 6 (100) | 7 (100) | 19 (100) |
| Diarrhea | 3 (100) | 3 (100) | 6 (100) | 6 (85.7) | 18 (94.7) |
| Aspartate aminotransferase increased | 3 (100) | 1 (33.3) | 4 (66.7) | 3 (42.9) | 11 (57.9) |
| Alanine aminotransferase increased | 2 (66.7) | 1 (33.3) | 3 (50.0) | 3 (42.9) | 9 (47.4) |
| Proteinuria | 1 (33.3) | 1 (33.3) | 2 (33.3) | 3 (42.9) | 7 (36.8) |
| Anemia | 1 (33.3) | 1 (33.3) | 1 (16.7) | 3 (42.9) | 6 (31.6) |
| Hyperphosphatemia | 0 | 0 | 2 (33.3) | 4 (57.1) | 6 (31.6) |
| Increased Blood Alkaline Phosphatase | 0 | 0 | 2 (33.3) | 3 (42.9) | 5 (26.3) |
| **Grade 5 TEAEs related to EVER4010001 and/or pembrolizumab** | 0 | 0 | 0 | 0 | 0 |
| **TEAEs leading to discontinuation of EVER4010001 and/or pembrolizumab** | 0 | 0 | 1 (16.7) | 3 (42.9) | 4 (21.1) |
| Investigations | 0 | 0 | 1 (16.7) | 2 (28.6) | 3 (15.8) |
| Aspartate aminotransferase increased | 0 | 0 | 0 | 2 (28.6) | 2 (10.5) |
| Blood bilirubin increased | 0 | 0 | 1 (16.7) | 0 | 1 (5.3) |
| Vascular disorders | 0 | 0 | 0 | 1 (14.3) | 1 (5.3) |
| Superior vena cava syndrome | 0 | 0 | 0 | 1 (14.3) | 1 (5.3) |
| **TEAEs resulting in temporary interruption of EVER4010001 and/or pembrolizumab** | 2 (66.7) | 1 (33.3) | 1 (16.7) | 1 (14.3) | 5 (26.3) |
| **TEAEs resulting in temporary interruption of EVER4010001** | 1 (33.3) | 1 (33.3) | 1 (16.7) | 1 (14.3) | 4 (21.0) |
| **TEAEs resulting in temporary interruption of pembrolizumab** | 1 (33.3) | 0 | 1 (16.7) | 1 (14.3) | 3 (15.8) |
| Endocrine disorders | 1 (33.3) | 0 | 0 | 0 | 1 (5.3) |
| Thyroiditis | 1 (33.3) | 0 | 0 | 0 | 1 (5.3) |
| Investigations | 0 | 0 | 1 (16.7) | 1 (14.3) | 2 (10.5) |
| Alanine aminotransferase increased | 0 | 0 | 0 | 1 (14.3) | 1 (5.3) |
| Blood bilirubin increased | 0 | 0 | 1 (16.7) | 0 | 1 (5.3) |
| **All SAEs** | 1 (33.3) | 1 (33.3) | 0 | 0 | 2 (10.5) |
| Gastrointestinal disorders | 1 (33.3) | 0 | 0 | 0 | 1 (5.3) |
| Pancreatitis | 1 (33.3) | 0 | 0 | 0 | 1 (5.3) |
| General disorders and administration site conditions | 0 | 1 (33.3) | 0 | 0 | 1 (5.3) |
| Death | 0 | 1 (33.3) | 0 | 0 | 1 (5.3) |
| Product issues | 1 (33.3) | 0 | 0 | 0 | 1 (5.3) |
| Device dislocation | 1 (33.3) | 0 | 0 | 0 | 1 (5.3) |

BID, twice daily; PT, preferred term; SOC, system organ class; TEAE, treatment-emergent adverse event
